# Decreasing expression of the G_1_-phase inhibitors, p21Cip1 and p16INK4a, promotes division of corneal endothelial cells from older donors

**Published:** 2010-05-25

**Authors:** Nancy C. Joyce, Deshea L. Harris

**Affiliations:** Schepens Eye Research Institute and Department of Ophthalmology, Harvard Medical School, Boston, MA

## Abstract

**Purpose:**

The current studies were conducted to determine whether the cyclin-dependent kinase inhibitors, p21Cip1 (p21 cyclin-dependent kinase-interacting protein 1) and p16INK4a (p16 cyclin-dependent kinase inhibitor 1A), help mediate G_1_-phase inhibition in human corneal endothelial cells (HCEC) by testing the effect of siRNA (small interfering RNA)-mediated down-regulation of the expression of these inhibitors on cell cycle entry and proliferation in HCEC cultured from older donors.

**Methods:**

HCEC were obtained from National Disease Research Interchange, Philadelphia, PA, and cultured according to published methods. Cells were electroporated in the presence of either a non-silencing siRNA control or p21+p16 siRNA. The efficiency of siRNA transfer was observed by fluorescence microscopy of Cy3-labeled control siRNA. Viability was determined by direct counting of cells before and after electroporation. The ability of p21+p16 siRNA to decrease the protein expression of p21Cip1 and p16INK4a was determined by semi-quantitative analysis of western blots. The effect of siRNA treatment on cell cycle progression and proliferation was determined 1, 5, and 11 days after electroporation by counting Ki67-positive cells and total DAPI-stained nuclei.

**Results:**

siRNA was efficiently transferred to HCEC by the electroporation method. The average cell loss was 41.25% at 24 h following electroporation. Protein levels of both p21Cip1 and p16INK4a were significantly decreased as the result of p21+p16 siRNA treatment. This treatment significantly increased the average number of Ki67-positive cells over controls and increased the total number of cells in a time-dependent manner.

**Conclusions:**

Both p21Cip1 and p16INK4a are involved in negative regulation of the cell cycle in HCEC and, thereby, provide an effective barrier to cell division. The siRNA-induced reduction in expression of these proteins increased the number of cells entering the cell cycle, as well as total cell numbers. Thus, reduction of the levels of p21Cip1 and p16INK4a could be useful in the development of treatments to induce transient cell division to increase corneal endothelial cell density.

## Introduction

Human corneal endothelial cells (HCEC) in vivo retain proliferative potential, although they do not normally divide as a means of tissue repair [1]. The ability of HCEC to divide has been demonstrated using ex vivo cornea model systems in which cell-cell contacts were released either by mechanical wounding of the endothelium [2] or by treatment of the endothelium with EDTA to release cell-cell junctions [3,4]. In both ex vivo model systems, HCEC entered the cell cycle and underwent cell division after mitogenic stimulation. HCEC are also able to divide in culture in the presence of appropriate mitogens [5,6]. Studies using both the ex vivo cornea wound models and cultured cells clearly indicate that the proliferative capacity of HCEC decreases in an age-dependent manner [2,6].

The endothelium plays a key role in maintaining corneal transparency. As such, it is important to find methods to increase the density of HCEC in patients at risk for vision loss due to low endothelial cell density. One important way to increase the density of HCEC is to take advantage of their capacity to divide. Current research goals related to the proliferative capacity of HCEC include: 1) Inducing in vivo cell division to repair corneal endothelium following trauma; 2) Increasing the density of HCEC in ex vivo corneas to be used for keratoplasty; and 3) Promoting division of HCEC in culture to provide a sufficient population of healthy, functional HCEC for tissue bioengineering. To achieve these goals, this laboratory has conducted studies to explore the molecular mechanisms that regulate proliferation of HCEC.

The cell cycle is divided into four distinct phases, leading to the formation of daughter cells. Studies have demonstrated that HCEC in vivo are inhibited in G_1_-phase of the cell cycle [7,8]. G_1_-phase is the initial portion of the cell cycle that occurs upon exposure to mitogens. During this phase, cells prepare for the process of DNA duplication, which occurs in S-phase. Cells will remain in G_1_-phase until all conditions required for normal DNA duplication have been met. Movement of cells into S-phase requires activation of the transcription factor, E2F, which regulates the expression of several proteins required for DNA duplication [9]. In the G_0_- (resting phase of the cell cycle) and early G_1_-phase, the retinoblastoma protein, Rb, tightly binds E2F, maintaining it in an inactivated state. Following mitogenic stimulation, the Rb protein becomes hyperphosphorylated by specific cyclin-dependent kinase complexes, resulting in the subsequent activation of E2F. Negative regulation of G_1_-phase is mediated, in part, by the activity of cyclin-dependent kinase inhibitors (CKIs). These inhibitors help prevent the hyperphosphorylation of Rb and subsequent activation of E2F [10-12]. The p27Kip1 (kinase inhibitor protein-1) inhibitor is expressed at relatively high levels in mitogen-starved cells [13] and helps mediate both contact-dependent and transforming growth factor-β-induced inhibition of proliferation [14]. The CKI, p21Cip1 (p21 cyclin-dependent kinase-interacting protein 1), is an important transcriptional target of the tumor suppressor, p53, and mediates both G_1_- and G_2_-phase checkpoint arrest in response to stresses, such as oxidative DNA damage [15,16]. Molecular studies have demonstrated an important role for p16INK4a (p16 cyclin-dependent kinase inhibitor 1A) in the development of cellular senescence [17]. Induction of p16INK4a expression, via various molecular biologic approaches, results in G_1_-phase arrest and development of a senescent phenotype [18,19]. Decreasing the expression of p21Cip1 or p16INK4a in knockout mice or in cells in which p21Cip1 or p16INK4a has been down-regulated by treatment with inhibitory peptides [18], antisense oligonucleotides [20], or small interfering RNA (siRNA) [21], can overcome negative G_1_-checkpoint regulation [22], promote a temporary escape from senescence [23], and induce cell proliferation [20].

Corneal endothelial cells in vivo express p27Kip1, p21Cip1, and p16INK4a [8,24]. Western blot analysis of HCEC isolated from young (≤30 years old) and older donors (≥50 years old) [25] indicates that there is no significant age-related difference in the relative expression of p27Kip1. Interestingly, treatment of confluent cultures of HCEC with p27Kip1 siRNA promoted proliferation of cells cultured from young, but not older donors [26]. This suggests that G_1_-phase in HCEC from older donors is negatively regulated by additional mechanisms. Western blot analysis of HCEC also revealed that the expression of both p21Cip1 and p16INK4a increases significantly with donor age, implicating these CKIs as mediators of the age-related decrease in proliferative capacity observed in these cells.

To develop methods to induce transient proliferation of HCEC, it is important to explore the molecular mechanisms involved in regulation of the cell cycle in HCEC from older donors. This is especially important because it is this segment of the population that normally has vision loss due to low endothelial cell density and it is also this segment of the population that provides the majority of donor corneas to be used for transplantation or for use in corneal tissue bioengineering. The current studies were conducted to determine whether p21Cip1 and p16INK4a help mediate G_1_-phase inhibition in HCEC by testing the effect of siRNA-mediated down-regulation of the expression of these inhibitors on cell cycle entry and proliferation in HCEC cultured from older donors.

## Methods

### Human donor corneas and culture of HCEC

Donor human corneas were obtained from National Disease Research Interchange (NDRI), Philadelphia, PA. Donor confidentiality was maintained by the eye bank, NDRI, and this laboratory according to the tenets of the Declaration of Helsinki. Table 1 presents relevant donor information and indicates the specific experiments the corneas were used for. Corneas were accepted for study based on published exclusion criteria [6]. Corneas were accepted only if the donor history and condition of the corneas indicated no damage to the health of the endothelium. Endothelial cell counts for all accepted corneas were at least 2,000 cells/mm^2^. HCECs were cultured following previously described protocols [5,6]. Briefly, Descemet’s membrane with the attached endothelium was carefully dissected from corneas in small strips and incubated overnight in OptiMEM-I (Invitrogen-Life Technologies, Carlsbad, CA) supplemented with 8% fetal bovine serum (FBS: Hyclone, Logan, UT) to stabilize the cells.  After gentle centrifugation, strips were incubated in 0.02% EDTA at 37 °C for 1 h to loosen cell-cell junctions. Cell junctions were mechanically disrupted by forcing the tissue and culture medium through the narrow opening of a flame-polished glass pipette. Cells were pelleted and then resuspended in culture medium. Culture medium consisted of OptiMEM I supplemented with 8% FBS, 5 ng/ml epidermal growth factor (EGF from mouse submaxillary gland; Upstate Biotechnologies, Lake Placid, NY), 100 μg/ml pituitary extract (Biomedical Technologies), 20 μg/ml ascorbic acid, 200 μg/ml calcium chloride, 0.08% chondroitin sulfate, antibiotic/antimycotic solution diluted 1/100 (all from Sigma-Aldrich, St. Louis, MO) and 50 μg/ml gentamicin (Invitrogen-Life Technologies). After passage 2, EGF and pituitary extract were removed from the medium. The majority of HCEC were passaged 2–4 times before use in these experiments.

**Table 1:** Donor Information.

**Donor age**	**Hours**	**Days**	**Cause of death**	**Experiment**
27	06:00	2	Pulmonary Edema	Western blot
28	14:00	3	Internal Bleeding/MVA	siRNA transfer/western
52	07:30	3	Ischemic Cardiomyopathy	Viability test
53	10:30	3	Myocardial Infarction	Viability test/Ki67 ICC
54	18:00	3	Myocardial Infarction	Ki67 ICC
57	07:00	3	Cardiac Arrest	Ki67 ICC
61	06:00	3	Breast Cancer	Viability test/Ki67 ICC
68	09:30	3	Cardiogenic Shock	Western blot
69	06:50	3	Myocardial Infarction	Western blot
71	07:00	3	Hypokalemia	Western blot
73	14:00	3	Heart Disease	Western blot
73	12:00	2	Electrocution	Viability test/Ki67 ICC
74	05:30	3	Myocardial Infarction	Viability test
77	11:00	3	Myocardial Infarction	Western blot

The “Hours” column indicates the number of hours between death and corneal preservation and the “Days” column indicates the number of days of corneal preservation in Optisol-GS at 4 °C.

### Electroporation of siRNA

Confluent HCEC were removed from the culture dish by treatment for 6–8 min with 0.05% trypsin-EDTA (Invitrogen-Life Technologies) at 37 °C. Trypsin activity was inhibited by addition of culture medium containing 8% FBS. The trypsinized cells were incubated for 3 min in the presence of trypan blue and counted using a Cellometer Auto T4 instrument (Nexcelom Bioscience LLC, Lawrence, MA). Analysis of the cell counts indicated an average viability of 87.4% following trypsinization of the cultured cells (data not shown). The number of viable cells obtained following trypsinization acted as a basis for calculation of the number of cells to be used for electroporation. An Amaxa Nucleofector II device (Lonza, Basel, Switzerland) and a Basic Nucleofector Kit for Primary Mammalian Endothelial Cells using the T-23 program were used for electroporation. Methods and reagents were based on Amaxa-recommended protocols. Three different p21Cip1 siRNAs and three p16INK4a siRNAs (all siRNAs from Applied Biosystems/Ambion, Austin, TX) were tested for their ability to effectively reduce protein expression as indicated by semi-quantitative analysis of western blots (see below). A non-silencing siRNA control (Silencer negative control #1 siRNA; Ambion 4611; Applied Biosystems/Ambion) was used for most experiments. Following preliminary studies, 1 μg siRNA per 5×10^5^ cells in 100 μl Basic Nucleofector Solution was found to be most efficient for electroporation of HCEC. Immediately following electroporation, culture medium (without EGF or pituitary extract), which was pre-warmed to 37 °C, was added to the cells. Cells were then plated as required for the specific experiment.

### Detection of siRNA transfer

HCEC were electroporated as above with 1 μg Silencer Cy3-labeled Negative Control #1 siRNA (Ambion AM4621; Applied Biosystems/Ambion) and plated in normal culture medium. At 24 h following electroporation, Cy3-labeled siRNA transfer was visualized using a Nikon Eclipse TS100 inverted microscope with an epi-fluorescence attachment and a Nikon Coolpix995 digital camera (Nikon Instruments, Melville, NY).

### Test of viability following electroporation

HCEC were grown to confluence and then removed from the culture dish by trypsin-EDTA treatment. Trypsinized cells were treated with trypan blue and counted using a Cellometer Auto T4 instrument, as described above. Equal numbers of viable cells were either not electroporated or electroporated in the presence of Silencer negative control #1 siRNA (Ambion 4611; Applied Biosystems/Ambion) as above. Cells were then plated in culture medium without EGF or pituitary extract and viability was determined 24 h later. To test for viability, cells were gently washed, then trypsinized, treated with trypan blue, and counted using the Cellometer. The percent difference in cell counts between the non-electroporated control and the electroporated sample was calculated in five separate experiments and the results averaged.

### Western blot detection of p21Cip1 and p16INK4a

HCEC were either not electroporated or electroporated in the presence of siRNA. Cells were then cultured for 72 h (in medium without EGF or pituitary extract) before protein extraction. Protein was extracted by incubating HCEC for 30 min at 4 °C in lysis buffer containing 1% Triton X-100, 250 mM NaCl, 2 mM EDTA, 50 mM Tris-HCl (pH 7.4), 10 µg/ml aprotinin, 10 µg/ml leupeptin, 1 mM phenylmethylsulfonyl fluoride, 50 mM sodium fluoride, and 0.1 mM sodium orthovanadate (all from Sigma), followed by homogenization and centrifugation. Equal concentrations of soluble protein were loaded on 10% Bis-Tris gels (Invitrogen-Life Technologies) for SDS–PAGE and then transferred to a polyvinylidene difluoride (PVDF) membrane (Millipore, Bedford, MA). The membrane was incubated overnight at 4 °C in p21Cip1 mouse monoclonal antibody diluted 1:2,000 (Cell Signaling, Danvers, MA), p16INK4a rabbit polyclonal antibody diluted 1:400 (Santa Cruz Biotechnology, Santa Cruz, CA) or beta-actin mouse monoclonal antibody diluted 1:3,000 (Sigma). All antibodies were diluted in 5% non-fat dry milk, plus 0.1% Triton X-100 in phosphate-buffered saline (PBS; Invitrogen-Life Technologies). Membranes were then rinsed 2 times for 10 min each with PBS containing 0.1% Triton X-100 and incubated 1 h at room temperature with HRP-conjugated donkey anti-mouse or anti-rabbit IgG (Jackson ImmunoResearch Laboratories, West Grove, PA) diluted at 1:5,000 in 5% non-fat dry milk in 0.1% Triton X-100/PBS. After washing the membranes 2 times for 10 min with PBS containing 0.1% Triton X-100, antibody binding was detected using a chemiluminescent substrate (SuperSignal West Pico or West Femto; Pierce, Rockford, IL). Membranes were then exposed to film to permit visualization of specific protein expression. Semi-quantitative analysis of protein expression was performed by densitometry using NIH Image software (NIH Image 1.34; National Institutes of Health, Bethesda, MD). Expression of p21Cip1 and p16INK4a was normalized relative to that of beta-actin.

### Assessment of the effect of p21+p16 siRNA treatment on cell cycle activity

HCEC cultured from the corneas of five older donors (>50 years old) were grown to confluence as above, removed from the tissue culture plate by treatment with trypsin/EDTA, then transferred to culture medium containing 8% FBS to inhibit the trypsin activity. Cell viability was determined using the Cellometer method described above. An average of 2.0×10^5^ cells/100 μl was used for each electroporated sample. Cells were electroporated with either Silencer negative control #1 siRNA or p21+p16 siRNA as above. Following electroporation, cells were seeded onto multiwell chamber slides and incubated in culture medium with 8% FBS, but without EGF or pituitary extract. At 1, 5, and 11 days after plating, cells were immunostained for Ki67, a marker of actively cycling cells [27]. At each time point, cells were washed quickly 3 times with PBS and then fixed with 100% methanol for 10 min at −20 °C. All subsequent steps were performed at room temperature. Cells were washed 3 times in PBS for 10 min each, permeabilized for 10 min with 1% Triton X-100 in PBS, and then washed 3 times in PBS for 10 min each. Non-specific binding was blocked using 4% BSA in PBS. Cells were incubated for 2 h in pre-diluted mouse monoclonal Ki67 antibody (Invitrogen-Life Technologies). Cells were washed 3 times in PBS for 10 min each and then incubated for 1 h in FITC-conjugated donkey anti-mouse IgG diluted at 1:200 in 4% BSA in PBS. The cells were washed in PBS 3 times for 10 min each and prepared in mounting medium containing 4’,6 diamidino-2-phenylindole (DAPI: Vector Laboratories, Burlingame, CA) to stain all nuclei. Digital fluorescent images were obtained using a Nikon Eclipse E800 microscope with VFM epi-fluorescence attachment (Nikon, Inc., Melville, NY) equipped with a Spot digital camera and Spot Advanced version 4.5 CE software (Diagnostic Instruments, Sterling Heights, MI). The number of Ki67-positive cells and total nuclei were counted in nine 20× magnification fields for each HCEC culture, time point, and treatment condition. The average number of cells, determined by counting DAPI-positive nuclei, and the average number of Ki67-positive cells were calculated and the results compared.

### Statistical analysis

Statistical analysis for both the western blot data and the Ki67/DAPI data was performed using the unpaired Student’s *t*-test. A p-value of ≤0.05 was considered to be statistically significant.

## Results

### Transfer efficiency and viability following electroporation

**Figure 1:**
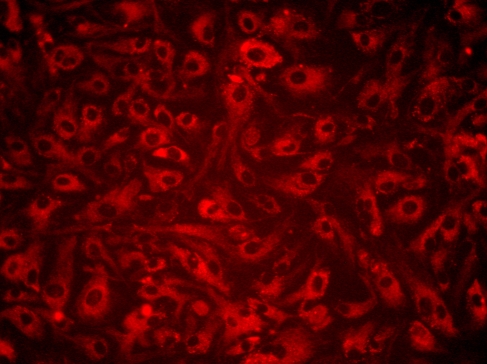
Evidence of highly efficient siRNA transfer. HCEC were electroporated with 1 μg Silencer Cy3-labeled Negative Control #1 siRNA and plated in culture medium as described in the “Methods” section. This representative image was taken 24 h after electroporation. Punctate cytoplasmic staining demonstrates the high efficiency of siRNA transfer to the cells. Original magnification: 20×.

Preliminary tests of transfer efficiency were conducted using different electroporation parameters. Electroporation of HCEC with Silencer Cy3-labeled Negative Control #1 siRNA, when conducted under the conditions described in the “Methods” section, yielded the best results. The representative fluorescence microscopic image presented in Figure 1 demonstrates the highly efficient transfer of Cy3-labeled siRNA to the cytoplasm of cells within the microscopic field. Counts of electroporated samples of HCEC cultured from five different donors compared with the corresponding non-electroporated samples indicate an average cell loss of 41.25% at 24 h following electroporation with a range of 27% to 66% (data not shown).

### Down-regulation of p21Cip1 and p16INK4a protein levels following siRNA treatment

**Figure 2:**
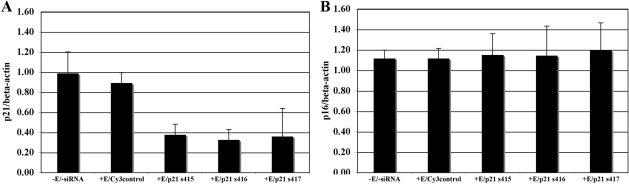
Effect of treatment with 3 different p21 siRNAs on p21Cip1 and p16INK4a protein levels. The graph of western blot results in (**A**) shows the effect of p21 siRNAs s415, s416, and s417 on the protein level of p21Cip1. The graph in (**B**) shows the effect of the same 3 p21 siRNAs on the protein level of p16INK4a. In the graphs, each bar shows the average relative expression of p21Cip1 or p16INK4a (+/− SEM) in HCEC cultured from 3 older donors. Beta-actin was used to normalize all results. Conditions included no electroporation or siRNA treatment (-E/-siRNA), electroporation with Silencer Cy3-labeled Negative Control #1 siRNA (+E/Cy3control), and electroporation with each of three siRNAs (+E/p21 s415, s416, or s417).

Preliminary western blot studies were conducted to determine the effect of three different p21 siRNAs (Ambion s415, s416, or s417) on the protein levels of both p21Cip1 and p16INK4a in cultured HCEC. As shown by the graphs in Figure 2, at 72 h after electroporation, all three p21 siRNAs significantly reduced the level of p21Cip1 compared with non-electroporated controls (p≤0.05) and with controls electroporated with Silencer Cy3-labeled Negative Control #1 siRNA (p≤0.02). None of the p21 siRNAs induced a significant change in the expression of p16INK4a (p=0.93 for all 3 siRNAs tested). These results demonstrate the specificity of the p21 siRNA treatment and provide evidence that lowering the protein level of p21Cip1 does not result in a compensatory increase in the level of p16INK4a. Three p16 siRNAs (Ambion s216, s217, s218) were similarly tested and all three efficiently reduced p16INK4a protein levels, but did not affect the expression of p21Cip1 (data not shown).

**Figure 3:**
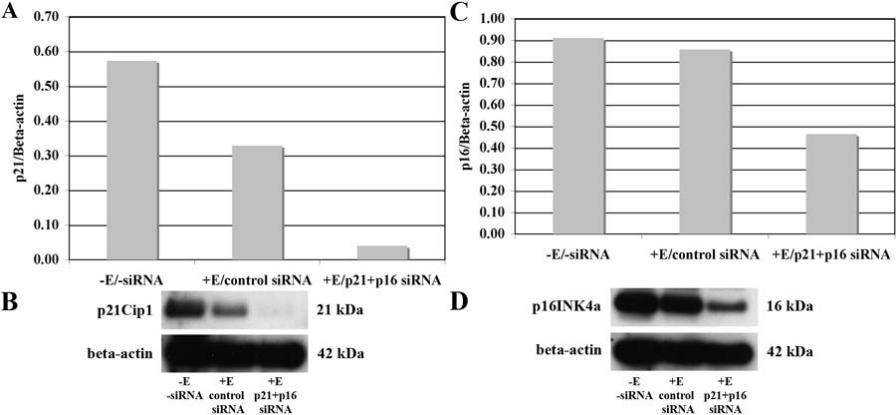
Representative example of the effect of treatment with p21+p16 siRNA on the protein levels of p21Cip1 and p16INK4a in HCEC cultured from a 71-year-old donor. **A**: Densitometric analysis of the effect of p21+p16 siRNA treatment on p21Cip1 protein expression in non-electroporated HCEC (-E/-siRNA), in HCEC electroporated with Silencer negative control #1 siRNA (+E/control siRNA), and in HCEC electroporated with p21+p16 siRNA (+E/p21+p16 siRNA). **B**: Western blots showing relative expression of p21Cip1 compared with beta-actin under each experimental condition. **C**: Densitometric analysis of the effect of p21+p16 siRNA treatment on p16INK4a protein expression under the same conditions as in **A**. **D**: Western blots showing relative expression of p16INK4a compared with beta-actin under each experimental condition. Results in **A** and **C** are expressed relative to beta-actin.

**Figure 4:**
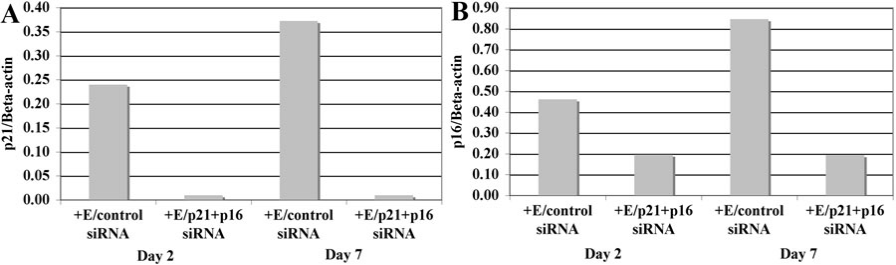
Effect of p21+p16 siRNA on p21Cip1 and p16INK4a protein expression 2 and 7 days following treatment. HCEC from a 68-year-old donor were electroporated with either control siRNA (+E/control siRNA) or p21+p16 siRNA (+E/p21+p16 siRNA). Western blots were used to analyze p21Cip1 (**A**) and p16INK4a (**B**) protein levels at the 2 time-points. Results are expressed relative to beta-actin. Note that both p21Cip1 and p16INK4a were decreased to similar levels at both time points.

A second series of studies tested the effect of combined treatment of HCEC with Ambion s416 p21 siRNA plus Ambion s217 p16 siRNA (p21+p16 siRNA). Cells were treated with both siRNAs, because previous work from this laboratory had indicated that both p21Cip1 and p16INK4a are expressed at significantly higher levels in HCEC from older donors, suggesting that, together, these two CKIs are responsible for mediating the reduced proliferative response of HCEC with increasing donor age [25]. HCEC cultured from three older donors were treated with p21+p16 siRNA and the effect on the protein expression of both CKIs was determined by western blot analysis. Results demonstrated that treatment of HCEC with p21+p16 siRNA consistently decreased the protein levels of both CKIs. Representative results from a 71-year-old donor are shown in Figure 3. The graphs in Figure 4 show representative western blot analyses of the relative protein levels of p21Cip1 and p16INK4a in HCEC from a 68-year-old donor 2 and 7 days after electroporation with control or p21+p16 siRNA. Results show similarly decreased levels of both p21Cip1 and p16INK4a at both time points, indicating that siRNA treatment remained effective in decreasing the expression of both CKIs for at least one week.

### p21+p16 siRNA treatment promotes cell cycle progression

**Figure 5:**
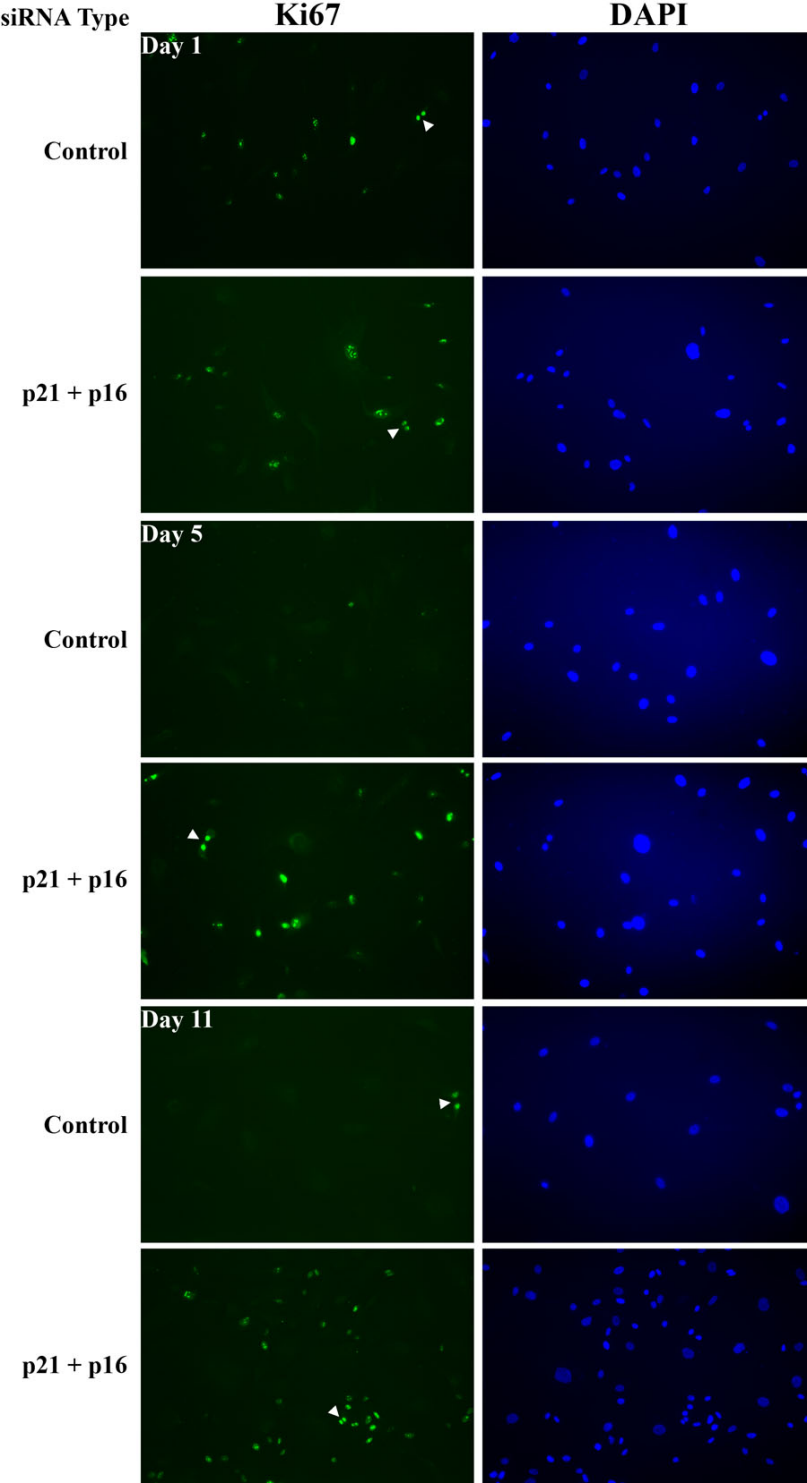
Representative fluorescence microscopic images of actively cycling HCEC from a 73-year-old donor. HCEC were electroporated with control or p21+p16 siRNA and then seeded onto multi-well chamber slides. On Days 1, 5, and 11 after electroporation, cells were immunostained for Ki67 (green) to view actively cycling cells and with DAPI (blue) to visualize all nuclei. Arrowheads indicate Ki67-positive dividing cells. Original magnification was 20×.

**Figure 6:**
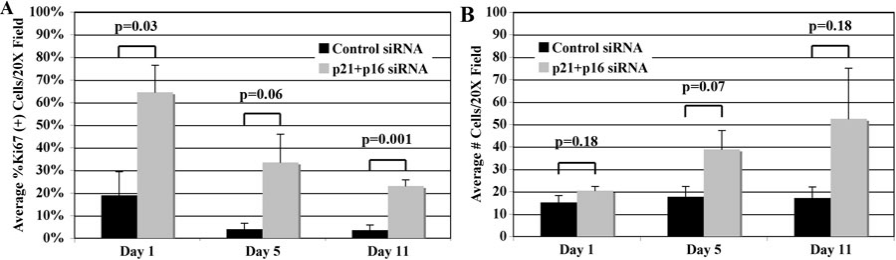
Effect of siRNA treatment on cell cycle entry and cell division. HCEC were treated with control or p21+p16 siRNA. On Days 1, 5, and 11, cells in the same 20× microscopic field were counted to determine the average percent of Ki67-positive cells, as an indication of the effect of treatment on cell cycle entry (**A**) and the average total number of DAPI-stained nuclei, as an indication of cell division (**B**). Data represents the average results from five different donors. Brackets on each bar represent SEM. P-values are indicated for each condition and time point.

The fact that p21+p16 siRNA treatment significantly down-regulated both CKIs compared with control siRNA provided a method to test whether p21Cip1 and p16INK4a proteins negatively regulate G_1_-phase of the cell cycle and whether treatment with p21+p16 siRNA would both promote cell cycle progression and increase cell numbers in HCEC from older donors. To test these ideas, HCEC from the each of five donors were treated with either p21+p16 siRNA or Silencer negative control #1 siRNA and then cultured in the presence of 8% FBS. On Days 1, 5, and 11 after electroporation, the average percent of Ki67-positive cells was determined as an indicator of actively cycling cells and average total cell numbers were determined by counting total DAPI-stained nuclei. Figure 5 shows representative images of Ki67-stained cells and corresponding DAPI-stained nuclei in HCEC from a 73-year-old donor treated with control or p21+p16 siRNA. Figure 6 presents the overall results. The bar-graph in Figure 6A compares the average percent of Ki67-positive HCEC per 20× field at each time point. An average of 19% of control cells had entered the cell cycle on Day 1 compared with 65% of cells treated with p21+p16 siRNA (p=0.03). Under both siRNA conditions, the average percent of Ki67-positive cells decreased over time; however, the percent of Ki67-positive cells in the p21+p16 siRNA-treated group was higher at each time point, strongly suggesting that down-regulation of the expression of p21Cip1 and p16INKa significantly increases cell cycle entry in HCEC from older donors. The decreasing number of Ki67-positive cells over the time-frame of the study suggests that there is an increase in the number of cells completing the cell cycle over that period of time. The bar-graph in Figure 6B compares the average number of cells present per 20× field for each time point and treatment condition based on counts of DAPI-stained nuclei. Ki67- and DAPI-stained cells were counted in the same microscopic fields. On Day 1, the average number of cells was similar, regardless of the siRNA treatment condition, indicating a similar plating efficiency under the two conditions. In cultures treated with control siRNA, the relative number of cells increased only slightly on Days 5 and 11, indicating that only a small percentage of cells had divided, thereby completing the cell cycle. Statistical analysis did not indicate a significant difference in total cell numbers between the control and p21+p16 siRNA-treated groups for any of the three time points, apparently due to the relatively high scatter in the data. However, average cell numbers in the p21+p16 siRNA-treated group increased 2.6× over the time of the study compared with an increase of only 1.1× for cells treated with control siRNA. Together, these results provide strong evidence that p21Cip1 and p16INK4a play an important role in negatively regulating G_1_-phase of the cell cycle in HCEC from older donors and that decreasing the protein expression of these CKIs by siRNA treatment not only promoted cell cycle entry, but also led to increased cell division.

## Discussion

Electroporation has been used by several investigators to transfer DNA plasmids to human corneal endothelial cells in tissue culture [28-30] and in organ culture [31], and in vivo to Wistar rats [32]. Electroporation was used in the current studies to promote efficient transfer of both p21 and p16 siRNA to primary HCEC. The specific method employed was the same as that reported by Engler et al. [28], who successfully transferred cDNA plasmids to primary cultures of HCEC. In preparation for the current studies, preliminary experiments were conducted to test corneal endothelial cell viability and efficiency using several Amaxa-based programs, including M03, T05, T23, T27, and U11. As was found by Engler et al. [28], the T23 program yielded the highest transfer efficiency. Although this method negatively affected cell viability, most cells survived the treatment, and were healthy enough to remain attached to the culture dish, to respond to siRNA treatment by down-regulating the expression of p21Cip1 and p16INK4a, and to respond to mitogenic stimulation by entering and completing the cell cycle to produce daughter cells.

The need to develop methods to increase HCEC density as an in vivo treatment, in ex vivo corneas to be used for keratoplasty, and in culture for corneal tissue bioengineering underlies the importance of identifying the molecular mechanisms that regulate the HCEC cell cycle. Although HCEC do not normally divide in vivo, the fact that they retain proliferative capacity suggests that it should be possible to find ways to induce transient proliferation in these cells based on the mechanisms that promote and/or inhibit their division. Importantly, studies have found that the relative proliferative capacity of HCEC decreases with donor age [2,6,33]. Western blot studies have suggested that this age-related difference in proliferative capacity results, at least in part, from the increased expression and activity of p21Cip1 and p16INK4a [25]. The current studies targeted HCEC from older donors to determine whether p21Cip1 and p16INK4a play important roles in the negative regulation of the cell cycle in these cells and to determine whether cell division could be enhanced in cells cultured from this age-group by down-regulating the expression of these inhibitors.

Results of these studies provide strong evidence that both p21Cip1 and p16INK4a are involved in negative regulation of the cell cycle in HCEC and provide an effective barrier to cell division. Results further indicate that siRNA-induced reduction in the expression of these proteins results in an increase in the number of cells entering the cell cycle, as well as in the number of cells completing the cycle, thereby increasing total cell numbers. Because HCEC were treated with a combination of siRNAs that specifically reduce the protein level of both p21Cip1 and p16INK4a, it is not possible to assess the relative contribution of each CKI to the inhibitory process. However, as indicated above, much is known about the activity of these inhibitors in other cell types and it is expected that they would have similar activities in HCEC, although the specific role of each CKI should still be investigated in these cells. These results also suggest that reduction of the levels of p21Cip1 and p16INK4a could be useful in the development of treatments to induce transient cell division to increase corneal endothelial cell density.
